# Immunogenicity and Safety of an Adjuvanted Herpes Zoster Subunit Vaccine Coadministered With Seasonal Influenza Vaccine in Adults Aged 50 Years or Older

**DOI:** 10.1093/infdis/jix481

**Published:** 2017-09-26

**Authors:** Tino F Schwarz, Naresh Aggarwal, Beate Moeckesch, Isabelle Schenkenberger, Carine Claeys, Martine Douha, Olivier Godeaux, Katrijn Grupping, Thomas C Heineman, Marta Lopez Fauqued, Lidia Oostvogels, Peter Van den Steen, Himal Lal

**Affiliations:** 1 Central Laboratory and Vaccination Centre, Klinikum Würzburg Mitte, Standort Juliusspital, Würzburg; 2 Gemeinschaftspraxis Dr Michael und Dr Beate Möckesch, Weinheim; 3 Klinische Forschung, Berlin, Germany; 4 Aggarwal and Associates, Brampton, Ontario, Canada; 5 GSK Vaccines, Wavre, Belgium; 6 Janssen Vaccines & Prevention, Leiden, the Netherlands; 7 GSK Vaccines, King of Prussia, Pennsylvania; 8 Present affiliation: Genocea Biosciences, Cambridge, Massachusetts; 9 Present affiliation: Pfizer Inc, Collegeville, Pennsylvania

**Keywords:** adjuvant, coadministration, herpes zoster, influenza, subunit vaccine

## Abstract

**Background:**

The immunogenicity and safety of an adjuvanted herpes zoster subunit (HZ/su) vaccine when coadministered with a quadrivalent seasonal inactivated influenza vaccine (IIV4) was investigated in a phase 3, open-label, randomized clinical trial in adults aged ≥50 years.

**Methods:**

Subjects were randomized 1:1 to receive either HZ/su (varicella zoster virus glycoprotein E; AS01_B_ Adjuvant System) and IIV4 at day 0 followed by a second HZ/su dose at month 2 (coadministration group), or IIV4 at month 0 and HZ/su at months 2 and 4 (control group). The primary objectives were the HZ/su vaccine response rate in the coadministration group and the noninferiority of the antibody responses to HZ/su and IIV4 in the coadministration compared with the control group. Safety information was collected throughout the duration of the study.

**Results:**

A total of 413 subjects were vaccinated in the coadministration group and 415 in the control group. The HZ/su vaccine response rate in the coadministration group was 95.8% (95% confidence interval, 93.3%–97.6%) and the anti–glycoprotein E GMC_Control/Coadmin_ ratio was 1.08 (.97–1.20). The primary noninferiority objectives were met. No safety concerns were observed.

**Conclusions:**

No interference in the immune responses to either vaccine was observed when the vaccines were coadministered, and no safety concerns were identified.

**Clinical Trials Registration:**

NCT01954251.

## Teaser

When the adjuvanted HZ subunit vaccine candidate was coadministered with a quadrivalent seasonal influenza vaccine, no interference in the immune responses were observed, and no safety concerns were identified.

**(See the editorial commentary by Oxman et al, on pages 1329–33 and major article Grupping et al, on pages 1343–51.)**

Reactivation of latent varicella-zoster virus (VZV) in sensory ganglia results in herpes zoster (HZ; shingles); typically a painful dermatomal rash that lasts for several weeks and can result in a neuropathic pain syndrome (postherpetic neuralgia) that can persist for months after the rash has resolved. The risk of HZ increases with age and is considered to be due to an age-dependent decline in VZV-specific cellular immunity. Risk is highest in adults >50 years of age, in whom 60% of all HZ cases occur [1–5].

Vaccination can reduce the risk of HZ. A live-attenuated HZ vaccine (Zostavax; Merck Sharp & Dohme) has demonstrated protective efficacy of 70% in adults aged 50–59 and 51% in those aged ≥60 years and is currently recommended in the United States for immunocompetent adults aged ≥60 years [2, 6–8]. A recombinant subunit HZ vaccine (HZ/su; GlaxoSmithKline [GSK]) consisting of VZV glycoprotein E (gE; 50 µg per dose) and the liposome-based adjuvant system AS01_B_ is currently under review for licensure in United States, Canada, and Europe [9–11]. HZ/su elicits strong anti-gE cellular and humoral responses and its protective efficacy in recent phase 3 clinical trials was 97.2% and 91.3% against HZ in adults aged ≥50 or ≥70 years, respectively, and 88.8% against postherpetic neuralgia in those aged ≥70 years [10, 11].

The risk of medical complications from influenza is also high in adults aged ≥50 years, for whom an annual seasonal influenza vaccination is recommended by public health authorities in the United States and by the World Health Organization [12, 13]. Quadrivalent seasonal influenza vaccines, which contain 2 influenza A strains (H1N1 and H3N2) and 2 influenza B-lineage strains (Victoria and Yamagata), may offer broader protection than trivalent vaccines against the influenza viruses circulating each year [14]. Inactivated split-virion influenza vaccines (IIVs) elicit humoral immune responses specific to the hemagglutinin protein of each vaccine strain. Although no immunologic correlate of protection has been definitively demonstrated, the protective role of these antibodies has been well established from human studies and from studies in experimentally infected animals [15, 16]. Because older adults could benefit from receiving HZ/su and influenza vaccinations in a single clinic visit, we investigated the immunogenicity, safety, and reactogenicity of a coadministration schedule in which the first dose of HZ/su is given with a licensed quadrivalent seasonal IIV (IIV4) in adults aged ≥50 years, compared with a sequential administration schedule.

## METHODS

### Study Design

This was a phase 3, randomized, open-label, multicenter clinical trial (Clinicaltrials.gov: NCT01954251) in adults aged ≥50 years conducted at 20 study centers in Canada (2 centers), Germany (15 centers), and the United States (3 centers). Subjects were stratified by age (50–59, 60–69, or ≥70 years) to ensure balanced representation of these age strata in the study, and then randomized 1:1 to 1 of the 2 parallel study arms using a central internet-based randomization system (GSK Vaccines). The coadministration group received the first dose of HZ/su and a dose of IIV4 in different arms at day 0 and the second dose of HZ/su at month 2. The control group received IIV4 at day 0, the first dose of HZ/su at month 2, and the second dose of HZ/su at month 4. Owing to the differences in vaccination schedules and in the vaccines themselves, the study was open label.

The primary objectives were to evaluate the vaccine response rate (VRR) to HZ/su 1 month after the second dose of the vaccine in the coadministration group, to demonstrate the noninferiority of anti-gE geometric mean concentrations (GMCs) after the second dose of HZ/su in coadministration versus control group, and to demonstrate the noninferiority of IIV4 immunogenicity in coadministration versus control groups for each vaccine strain by comparing the geometric mean titers (GMTs) of hemagglutination inhibition (HI) antibodies. Secondary objectives were to assess the noninferiority of HI antibody seroconversion rates (SCRs) in the coadministration group for each IIV4 strain versus those in control group, to assess IIV4 immunogenicity for each strain in terms of GMT and in terms of the age group–specific (age 50–64 or ≥65 years) Center for Biologics Evaluation and Research (CBER) criteria for seroprotection rates (SPRs) and SCRs [17], and to evaluate the safety and reactogenicity of both vaccines when coadministered or sequentially administered.

The study was conducted according to the International Conference on Harmonisation Guideline for Good Clinical Practice and the Declaration of Helsinki and was approved by all applicable national health regulatory agencies and any national, regional, or investigational center ethics committees or institutional review boards. All subjects provided written informed consent before participating in the trial.

### Study Participants

Adults aged ≥50 years were eligible to participate in the study. They were excluded if they had taken (or planned to take) any investigational or nonregistered drug or vaccine, or any nonstudy vaccine, from 30 days before study inclusion through 30 days after the second dose of HZ/su, had received influenza vaccine or had received long-term treatment with immunosuppressant drugs or immune-modifying drugs within 6 months before study inclusion, had received a previous VZV or HZ vaccination, or had a history of HZ.

### Vaccines

The HZ/su vaccine candidate (GSK1437173A, GSK Vaccines) contained 50 µg VZV gE and the AS01_B_ Adjuvant System containing 50 μg of MPL (3-O-desacyl-4’-monophosphoryl lipid A; produced by GSK) and 50 μg of QS-21 (*Quillaja saponaria* Molina, fraction 21; licensed by GSK from Antigenics, a wholly owned subsidiary of Agenus) and liposomes per 0.5 mL of reconstituted vaccine. The IIV4 (Influsplit Tetra in Germany, Fluarix Quadrivalent in Canada and the United States; GSK Vaccines) contained 15 µg of hemagglutinin from each of 4 strains (Northern Hemisphere formulation for 2013–2014) per 0.5-mL monodose syringe. The 4 strains were A/Christchurch/16/2010 (H1N1) NIB-74XP (an A/California/7/2009 [H1N1]-like strain), A/Texas/50/2012 (H3N2)/NYMC X-223A (antigenically similar to the cell-propagated prototype strain A/Victoria/361/2011 [H3N2]), B/Massachusetts/02/2012-(B/Yamagata lineage) NYMC BX-51B, and B/Brisbane/60/2008 (B/Victoria lineage).

### Outcomes and Assessments

Humoral immune responses to the vaccines were assessed from blood samples collected from the coadministration group at day 0 (prevaccination for both vaccines), day 21 (after vaccination for IIV4), and month 3 (1 month after the second dose of HZ/su); and from samples collected from the control group at day 0 (prevaccination for IIV4), day 21 (after vaccination for IIV4), month 2 (before vaccination for HZ/su), and month 5 (1 month after the second dose of HZ/su). Anti-VZV gE antibody concentrations were determined using an anti-gE enzyme-linked immunosorbent assay with a cutoff of 97 mIU/mL. A standard HI assay was used to determine the HI titer for each strain in IIV4 with a lower limit cutoff dilution of 1:10. All assays were performed by GSK Vaccines laboratories in Rixensart, Belgium, or Dresden, Germany.

### Safety and Reactogenicity

Diary cards were provided to subjects at each vaccination to collect the solicited and unsolicited adverse events (AEs). Solicited AEs were collected within 7 days after vaccination. Solicited local reactions were injection site pain, redness, and swelling; solicited general reactions were arthralgia, fatigue, fever, gastrointestinal symptoms (nausea, vomiting, diarrhea, abdominal pain), headache, myalgia, and shivering. Unsolicited nonserious AEs were collected during the 30 days after each vaccination. Serious AEs (SAEs) and potential immune-mediated diseases (pIMDs) were collected from day 0 through 12 months after the second dose of HZ/su. A suspected case of HZ was defined as a new rash characteristic of HZ and diagnosed by the investigator. HZ and HZ complications were collected until the end of the study.

### Statistical Analysis

Two main subject cohorts were defined. The total vaccinated cohort included all subjects who received ≥1 dose of any study vaccine. The according-to-protocol cohort for immunogenicity included subjects who received ≥1 dose of study vaccine, met all eligibility criteria, and had no major protocol deviations and for whom immunogenicity end-point results were available. The primary analysis of immunogenicity was based on the according-to-protocol cohort for immunogenicity; the analysis for safety was based on the total vaccinated cohort.

#### Primary Objectives

The objective for the VRR to HZ/su was met if the lower limit of the 2-sided 95% confidence interval (CI) of the VRR in the coadministration group was ≥60%. Noninferiority of the coadministration group versus the control group in terms of anti-gE GMCs was demonstrated if the upper limit of the 2-sided 95% CI of the postvaccination GMC_Control_/GMC_Coadmin__adjusted_ ratio was below a predefined limit of 1.5. Adjusted least squares means and differences of least squares means between the groups were calculated together with 2-sided 95% CIs and back-transformed to the original units to provide GMCs and GMC ratios. Postvaccination anti-gE GMCs at month 3 for the coadministration group and month 5 for the control group were adjusted according to the means of the prevaccination log-transformed anti-gE antibody concentrations (month 0 for the coadministration and month 2 for the control group). Noninferiority of HI antibody GMTs at postvaccination day 21 was demonstrated if the upper limit of the 2-sided 95% CI for the adjusted GMT ratio of control to coadministration group was <1.5 for each strain included in IIV4.

#### Secondary Objectives

The CBER criteria for noninferiority of the coadministration group versus the control group in terms of IIV4 HI SCRs for each strain was reached if the upper limit of the 95% CI of the SCR_Control_ – SCR_Coadmin_ differences at 21 days after vaccination was <10% [17]. IIV4 immunogenicity was assessed from the GMTs, SPRs, and SCRs for each strain at postvaccination day 21 and according to age group–specific CBER criteria (age 50–64 or and ≥65 years) for the SCRs and SPRs [17]. Safety parameters were analyzed using descriptive statistics. The incidence and intensity of each symptom was calculated with an exact 95% CI for each group. Statistical analyses were performed using Statistical Analysis Systems software (SAS; version 9.2).

#### Sample Size Calculations

With 393 evaluable subjects in each group, the overall power to meet all primary objectives was 93.7%. For each secondary objective considered separately, the nominal power was ≥80%. Assuming that about 5% of the subjects enrolled would be nonevaluable owing to dropout at day 21 after IIV4 vaccination, the enrollment target was set at 414 subjects per group. The sample size was not adjusted to have adequate power to demonstrate the primary objectives together with all secondary objectives. Therefore, the evaluation of secondary objectives was descriptive and should be considered with caution.

## RESULTS

**Figure 1.**
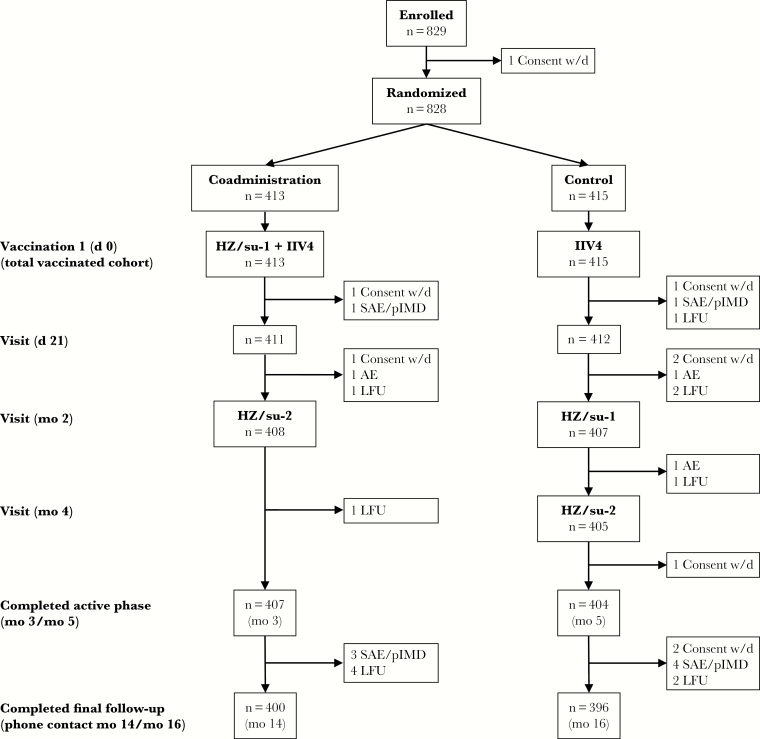
Subject disposition. A total of 828 subjects aged ≥50 years were randomized to the 2 treatment groups. Subjects in the coadministration group received the first herpes zoster subunit (HZ/su) vaccination (HZ/su-1) and the quadrivalent seasonal inactivated influenza vaccine (IIV4) vaccination in different arms on day 0 (d 0) and the second HZ/su vaccination (HZ/su-2) at month 2 (mo 2). Subjects in the control group received the IIV4 vaccination on day 0, HZ/su-1 at month 2, and HZ/su-2 at month 4. Blood samples for immunogenicity analyses were collected on day 0, day 21, and month 3 for the coadministration group, and on day 0, day 21, month 2, and month 5 for the control group. The final study follow-up was a phone call to collect final safety data 1 year after the HZ/su-2 vaccination. Subjects were withdrawn from the study owing to a serious adverse event (SAE) or potential immune-mediated disease (pIMD) (9 subjects), a nonserious adverse event (AE; 3 subjects), withdrawn consent (w/d; 8 subjects), or loss to follow-up (LFU; 12 subjects).

The study was conducted between 3 October 2013 and 20 March 2015, and 829 subjects were enrolled (Figure 1). A total of 828 subjects were vaccinated at least once with any study vaccine (total vaccinated cohort: 413 coadministration, 415 control), of whom 781 (386 in the coadministration and 395 in the control group) were included in the according-to-protocol cohort for immunogenicity. A total of 796 subjects (400 in the coadministration and 396 in the control group) completed the study. Subject demographics were similar for both groups (Table 1). Subjects were mostly of white/European ancestry with a mean age of 63.4 years in each group and similar sex ratios in the 2 groups. Approximately 60% of subjects in each group had received a seasonal influenza vaccination in the previous season.

**Table 1.** Subject Demographics

Characteristic	Subjects, No. (%)^a^	
	Control Group (n = 415)	Coadministration Group (n = 413)
Age, mean (standard deviation), y	63.4 (8.8)	63.4 (8.3)
Age group		
50–59 y	154 (37.1)	150 (36.3)
60–69 y	158 (38.1)	157 (38.0)
≥70 y	103 (24.8)	106 (25.7)
Sex		
Female	218 (52.5)	211 (51.1)
Male	197 (47.5)	202 (48.9)
Geographic ancestry		
African/African American	5 (1.2)	9 (2.2)
Asian/Southeast Asian	20 (4.8)	17 (4.1)
White/European	381 (91.8)	381 (92.3)
Other	9 (2.2)	6 (1.4)
Received influenza vaccination in previous season (2012–2013)		
Yes	254 (61.2)	249 (60.3)
No	161 (38.8)	164 (39.7)

^a^Values shown are for the total vaccinated cohort and represent No. (%) of subjects unless otherwise specified.

### Immunogenicity

The VRRs for anti-gE antibody concentrations were similar between the control (97.9%; 95% CI, 96.0%–99.1%) and the coadministration group (95.8%; 93.3%–97.6%) group (Table 2), and the VRR in the coadministration group met the first primary objective (lower limit of 95% CI, >60%). The GMC_Control_/GMC_Coadmin__adjusted_ ratio 1 month after the second dose of HZ/su was 1.08 (95% CI, .97–1.20), demonstrating that the coadministration group was noninferior to the control group, thus meeting the second primary objective. GMTs after IIV4 vaccination were similar for both study groups. GMT_Control_/GMT_Coadmin__adjusted_ ratio for all 4 strains were between 0.98 and 1.07, with the upper limit of all 95% CIs <1.5, demonstrating that the coadministration group was noninferior to the control group for all 4 of the IIV4 GMTs and meeting the third primary objective.

**Table 2.** HZ/su and IIV4 Immunogenicity Results and Analyses^a^

Objective	Control Group	Coadministration Group	Noninferiority Analysis
Primary objectives			
HZ/su VRR (95% CI), %^b^	n = 388	n = 382	…
	97.9 (96.0–99.1)	95.8 (93.3–97.6)	ND
Noninferiority of anti-gE GMC_Coadmin_ (95% CI), mIU/mL	n = 388	n = 382	GMC_Control_/GMC_Coadmin_ (95% CI)^c^
GMC	56848 (53 598–60 295)	52 861 (48 386–57 749)	ND
Adjusted GMC^c^	56 247 (52 177–60 635)	52 152 (48 356–56 245)	1.08 (0.97–1.20)^d^
Noninferiority of influenza GMT by strain (95% CI), 1/dilution	n = 394	n = 384	GMT_Control_/GMT_Coadmin_ (95% CI)^c^
GMT			
A/H1N1	193.2 (170.2–219.4)	196.2 (172.2–223.5)	ND
A/H3N2	66.8 (60.4–74.0)	65.4 (59.0–72.5)	ND
B/Victoria	185.2 (168.0–204.1)	177.2 (161.6–194.2)	ND
B/Yamagata	423.3 (388.0–461.8)	433.7 (401.3–468.7)	ND
Adjusted GMT^c^			
A/H1N1	194.3 (173.0–218.1)	187.5 (166.7–210.8)	1.04 (0.88–1.22)^d^
A/H3N2	65.9 (60.3–72.0)	63.7 (58.3–69.7)	1.03 (0.91–1.17)^d^
B/Victoria	181.6 (166.7–197.8)	170.2 (156.1–185.6)	1.07 (0.95–1.20)^d^
B/Yamagata	413.9 (383.4–446.8)	423.5 (392.0–457.5)	0.98 (0.88–1.09)^d^
Secondary objectives			
Noninferiority of influenza SCRs by strain (95% CI), %^e^	n = 394	n = 384	SCR_Control_ – SCR_Coadmin_ (95% CI)^f^
A/H1N1	60.9 (55.9–65.8)	60.4 (55.3–65.3)	0.50 (−6.36 to 7.35)^d^
A/H3N2	35.3 (30.6–40.2)	35.4 (30.6–40.4)	−0.14 (−6.85 to 6.57)^d^
B/Victoria	42.9 (37.9–47.9)	37.2 (32.4–42.3)	5.65 (−1.24 to 12.49)
B/Yamagata	37.6 (32.8–42.6)	40.1 (35.2–45.2)	−2.54 (−9.37 to 4.31)^d^

Abbreviations: CI, confidence interval; Coadmin, coadministration; gE, glycoprotein E; GMC, geometric mean concentration (anti-gE antibody); GMT, geometric mean titer (hemagglutination inhibition); HZ/su, herpes zoster subunit; ND, not done; SCR, seroconversion rate; VRR, vaccine response rate.

^a^Values shown are for the according-to-protocol immunogenicity cohort, for subjects with both pre- and postvaccination results available.

^b^A VRR to the HZ/su vaccine was defined as a postvaccination anti-gE antibody concentration that was either ≥4 fold the assay cutoff value (97 mIU/mL) for initially seronegative subjects or ≥4 fold the prevaccination antibody concentration for initially seropositive subjects 1 month after the second dose of HZ/su.

^c^The 95% CIs of anti-gE GMC and influenza GMT ratios were obtained using an analysis of covariance (ANCOVA) model on the log- transformed concentrations. This model included the vaccine group and age stratum (50–59, 60–69, or ≥70 years) as the fixed effect and the prevaccination log-transformed concentration or titer as the regressor. GMC and GMT ratios and their 95% CIs were derived as exponential transformations of the corresponding group contrast in the model. The 95% CIs for the adjusted GMT and GMC were obtained by exponential transformation of the 95% CIs for the group least squares mean of the above ANCOVA model.

^d^Coadministration group noninferior to control group.

^e^Seroconversion was defined as a postvaccination hemagglutination inhibition antibody titer of either ≥40 1/dilution for initially seronegative subjects or ≥4-fold the prevaccination antibody titer for initially seropositive subjects.

^f^SCR differences in which the 95% CI is the standardized asymptotic 95% CI.

**Figure 2.**
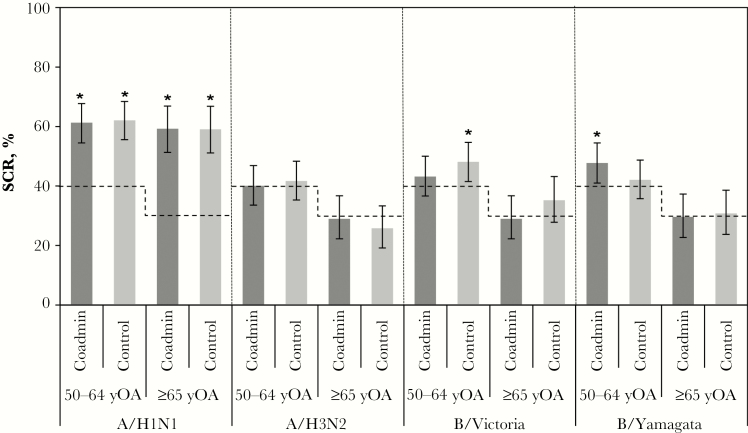
Seroconversion rates (SCRs) for the quadrivalent seasonal inactivated influenza vaccine (IIV4) according to age group and Center for Biologics Evaluation and Research (CBER) criteria. SCRs at postvaccination day 21 are presented for each age group in the coadministration (Coadmin) and control treatment groups in the according-to-protocol immunogenicity cohort for each influenza strain in IIV4. Seroconversion was defined as a postvaccination hemagglutination inhibition antibody titer of either ≥40 1/dilution for initially seronegative subjects or ≥4-fold the prevaccination antibody titer for initially seropositive subjects. Dotted lines represent minimum CBER-specified requirements for the lower limit of the 95% confidence interval (CI) of the SCR in each age group: 40% for subjects aged 50–64 years and 30% for those aged ≥65 years. Asterisks denote SCRs that met these criteria, and error bars represent 95% CIs.

**Figure 3.**
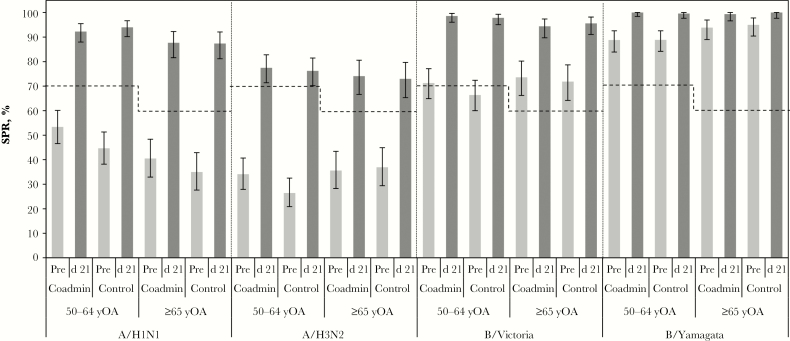
Seroprotection rates (SPRs) according to age group and Center for Biologics Evaluation and Research (CBER) criteria. SPRs at prevaccination (Pre) and postvaccination day 21 (d 21) are presented for subjects aged 50–64 or ≥65 years in the coadministration (Coadmin) and control treatment groups in the according-to-protocol immunogenicity cohort for each influenza strain in the quadrivalent seasonal inactivated influenza (IIV4) vaccine. Seroprotection was defined as a hemagglutination inhibition antibody titer ≥40 1/dilution. Dotted lines represent minimum CBER-specified requirements for the lower limit of the 95% confidence interval (CI) of the SPR in each age group: 70% for subjects aged 50–64 and 60% for those aged ≥65 years. All SPRs at day 21 met the age-specific CBER criteria. Error bars represent 95% CIs.

Overall, IIV4 SCRs were between 35% and 61% for each strain in both treatment groups, with the coadministration group noninferior to the control group for H1N1, H3N2, and B/Yamagata but not for B/Victoria (Table 2). CBER SCR criteria (Figure 2) were met for the following strains: H1N1 in both treatment and age groups (50–64 or ≥65 years), B/Victoria in the control group (age 50–64 years), and B/Yamagata in the coadministration group (age 50–64 years). CBER SCR criteria were not met for the H3N2 strain. Postvaccination influenza SPRs were comparably high in both treatment groups (>90% for the H1N1, B/Victoria, and B/Yamagata strains and about 75% for the A/H3N2 strain) and met all CBER criteria (Figure 3). Prevaccination SPRs were also relatively high in both treatment groups and both age groups, especially for the B/Victoria and B/Yamagata strains (Figure 3). In a post-hoc analysis performed to assess the impact of influenza vaccination in the previous season on IIV4 immunogenicity, postvaccination SPRs were similar for participants regardless of vaccination history (Supplementary Figure S1) and SCRs tended to be higher in subjects who did not have an influenza vaccination in the previous season (Supplementary Figure S2).

### Safety and Reactogenicity

**Figure 4.**
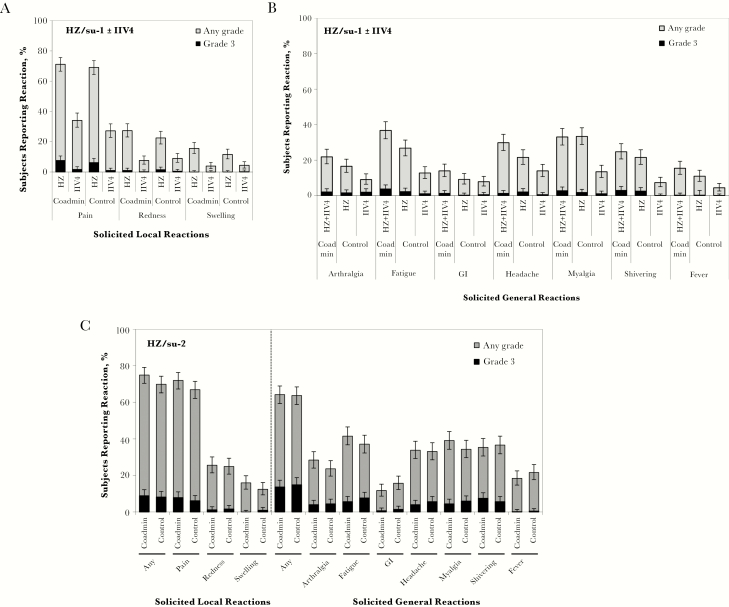
Reactogenicity of the herpes zoster subunit (HZ/su) and quadrivalent seasonal inactivated influenza (IIV4) immunizations. Solicited local and general reactions are presented for the total vaccinated cohort. The coadministration (Coadmin) group received the first dose of HZ/su and the IIV4 vaccine on day 0 and the second dose of HZ/su at month 2. The control group received the IIV4 vaccine on day 0, the first dose of HZ/su at month 2, and the second dose of HZ/su dose at month 4. *A,* Local reactions occurring in the coadministration group within 7 days after coadministration of the first dose of HZ/su (HZ) and IIV4 or in the control group within 7 days after each vaccine was administered separately are shown for each arm. Reactions for the coadministration group were recorded concurrently for 7 days after day 0; reactions for the control group were recorded for 7 days after day 0 for IIV4 and 7 days after the first dose of HZ/su was administered at month 2. *B,* General reactions occurring within 7 days after the first dose of HZ/su and IIV4 coadministration in the coadministration group or within 7 days after each vaccine was administered separately in the control group. General reactions for the coadministration group were recorded for 7 days after day 0 and were attributable to both vaccines given at the same time; reactions for the control group were recorded for 7 days after day 0 for IIV4 and 7 days after the first dose of HZ/su was administered at month 2, and so were attributable to each vaccine given separately. GI, gastrointestinal symptoms. *C,* General reactions occurring within 7 days after administration of the second dose of HZ/su in each group. Reactions were recorded in month 2 for the coadministration group and in month 4 for the control group. A local reaction for redness or swelling was recorded if the diameter was ≥20 mm; it was recorded as grade 3 intensity if the diameter was >100 mm. Fever was recorded if the oral temperature was ≥37.5°C; it was recorded as grade 3 intensity if it was >39.0°C. Other general reactions were recorded if they were mild or easily tolerated (no interference in normal daily activity), moderate (discomfort that interfered with normal daily activity), or severe (grade 3; significant discomfort that prevented normal daily activity). Error bars represent 95% confidence intervals.

Solicited local reactions of any type were reported by 79.3% of subjects when HZ/su and IIV4 were coadministered (10.0% reported grade 3), 72.3% after the first dose of HZ/su alone in the control group (7.4% reported grade 3), and 30.6% after receipt of IIV4 alone in the control group (1.5% reported grade 3). In general, incidences of specific solicited local reactions tended to be similar whether the vaccines were administered together or separately, although there were some minor differences (Figure 4A). Most local reactions were grade 1 or 2, and most resolved within 4 days. The most frequently reported local reaction after either vaccination was injection site pain, which was reported by approximately 70% of the subjects in each group after the first dose of HZ/su, and by 34.1% and 27.1% of the subjects in the coadministration and control groups, respectively, after IIV4 vaccination. Solicited local reactions of any type tended to be more frequent with HZ/su than with IIV4 in both groups.

Solicited general reactions of any type were reported by 60.9% of subjects when HZ/su and IIV4 were coadministered (8.8% reported grade 3), 52.1% after the first dose of HZ/su alone in the control group (5.7% reported grade 3), and 33.6% after receipt of IIV4 alone in the control group (2.7% reported grade 3). Solicited general reaction rates seemed slightly higher when HZ/su was coadministered with IIV4 than when it was administered alone (Figure 4B). Fatigue, myalgia, headache, and shivering were the most common general reactions after the first dose of HZ/su, whether it was administered with or without IIV4. Headache, myalgia, and fatigue were the most common general reactions to IIV4 alone. Most solicited general reactions to all vaccinations in both groups were either grade 1 or 2, and most resolved within 4 days. Overall, local and general reactogenicity was higher for HZ/su than for IIV4. After the second dose of HZ/su, local and general reaction incidences were similar in the 2 groups (Figure 4C).

Unsolicited AEs were reported for 110 subjects (26.6%; 194 AEs) in the coadministration and 162 subjects (39.0%; 295 AEs) in the control group. Of these, 23 AEs in the coadministration group (18 subjects; including grade 3 tinnitus in 1) and 30 AEs in the control group (26 subjects, including one subject with grade 3 injection site warmth and one subject with grade 3 night sweats) were considered to be vaccination related by the investigator. Sixty-three SAEs, 4 pIMDs (myasthenia gravis, rheumatoid arthritis, seventh nerve paralysis, and psoriasis), and 3 deaths occurred in the coadministration group; 60 SAEs, 2 pIMDs (vocal cord paralysis and ulcerative colitis) and 5 deaths occurred in the control group. All fatal events except 1 (metastatic hepatocellular cancer) occurred >90 days after the second dose of HZ/su. No SAEs, pIMDs, or deaths were considered to be vaccination related by the investigator. Three suspected cases of HZ were reported during the study. One occurred in the coadministration group 238 days after the second dose of HZ/su, and 2 occurred in the control group after IIV4 but before HZ/su vaccination.

## DISCUSSION

This study demonstrated that the immunogenicity of 2 doses of HZ/su was unaffected by coadministration of the first dose with a seasonal influenza vaccination and that immunogenicity of the seasonal influenza vaccine was not substantially affected by coadministration with HZ/su. The study met all primary immunogenicity objectives for overall HZ/su VRR in the coadministration group, and those for noninferiority of the immune responses to HZ/su and to IIV4 for coadministration compared with sequential administration. The study also met most secondary objectives, because coadministration of HZ/su and IIV4 resulted in noninferior SCRs for 3 of the 4 influenza strains, and postvaccination SPRs were all above the minimum CBER criteria. In addition, coadministration of HZ/su and IIV4 had no clinically meaningful effect on the local reactogenicity of either vaccine, though some general reactions seemed more frequent with coadministration. No safety concerns associated with vaccine coadministration were identified.

This is the first study to demonstrate that the immunogenicity of HZ/su is preserved when HZ/su is coadministered with another vaccine. The observed VRR of 95.8% in the coadministration group was similar to what has been observed in 2 pivotal phase 3 studies reporting a VRR of 98.5% and 95.9% in subjects aged ≥50 or ≥70 years, respectively (unpublished data). Vaccine efficacies against HZ in these studies were 97.2% in subjects aged ≥50 years and 91.3% in those aged ≥70 years across the 2 studies [10, 11]. No generally accepted immunologic correlate of protection has been established for HZ. Nonetheless, the observation that HZ/su humoral immunogenicity is unaffected by coadministration of its first dose with IIV4 provides confidence that HZ/su efficacy should not be affected by concurrent IIV4 vaccination.

The magnitude of the humoral responses to IIV4 is not affected by coadministration with HZ/su; at 21 days after vaccination, noninferiority of GMTs for each strain was met. SPRs for each strain were high and above the minimum CBER age-group criteria in both treatment groups. However, not all of the SCRs in both treatment groups met CBER criteria. This is not unexpected given the tendency for older adults to have high baseline HI antibody titers, arising from either natural exposure to influenza or previous vaccinations, which have been reported to reduce antibody responses to subsequent seasonal influenza vaccination [18–20]. In populations with high prevaccination SPRs, such as those seen in our study, it is difficult to demonstrate an improved immune response based on SCRs alone [21]. In addition, the immune responses to influenza vaccines are often less robust in older adults owing to immunosenescence [22–24]. In support of this possibility, IIV4 SCRs were higher in subjects who had not received an influenza vaccination in the previous season than in those who had. Importantly, post–IIV4 vaccination SPRs in both treatment groups met all CBER criteria, whether or not subjects had received the HZ/su vaccine at the same time.

These findings are in agreement with those of other studies evaluating coadministration of trivalent IIV with a second vaccine [25, 26]. Although antibody titers induced by either coadministered vaccine were in some cases slightly lower than with sequential administration, both administration regimens were considered equivalent with respect to immunogenicity and safety [25, 26].

Solicited local reactions after coadministration of HZ/su and IIV4 were generally similar in incidence and severity to those for each vaccine given separately. Systemic reactions to each of the coadministered vaccines cannot be distinguished. Consistent with the higher overall dose of vaccine immunogens from coadministration of the 2 vaccines, we observed that the incidences of most solicited general reactions tended to be higher for coadministered vaccines than for either vaccine administered alone. Furthermore, the incidences of grade 3 reactions in both groups were similar, suggesting that any increase in systemic reactogenicity due to coadministration has a limited clinical impact. In addition, none of the subjects in the coadministration group withdrew owing to a vaccine-related AE, the incidences of SAEs (including fatalities) and pIMDs were similar in both groups, and no AEs, SAEs, or pIMDS were assessed to be causally related to vaccination.

A strength of the study was that the participants were older adults, the population most in need of HZ and seasonal influenza vaccination and in whom efficacy data for HZ/su were generated. Moreover, a high proportion of subjects in each treatment group (>93%) completed the study according to protocol. A limitation was that the youngest adults in the study (aged 50–64 years) were at the high end of the age bracket for adult CBER criteria (18–64 years), an age at which immunosenescence may begin to affect immune responses to vaccines; this may have contributed to the failure of the IIV4 immunogenicity to meet certain CBER criteria in subjects aged 50–64 years. Another possible limitation is that the study was open label; however, this was required in practice owing to the differences in vaccination schedule for HZ/su and IIV4. Finally, secondary objectives were only descriptive, so their interpretation should be considered with caution.

In conclusion, coadministration of the HZ/su and IIV4 was well tolerated and did not affect overall GMTs for either vaccine, producing humoral responses that were noninferior to those produced by separate, sequential administration. Vaccine coadministration also did not raise any safety concerns. These results support the concomitant administration of HZ/su and seasonal influenza vaccine, which could benefit older adults by increasing opportunities to provide vaccination against HZ and influenza in a single clinic visit.

## Supplementary Data

Supplementary materials are available at *The Journal of Infectious Diseases* online. Consisting of data provided by the authors to benefit the reader, the posted materials are not copyedited and are the sole responsibility of the authors, so questions or comments should be addressed to the corresponding author.

## Supplementary Material

Supplementary Figure S1

Supplementary Figure S2

Patient Highlight Section

## Notes

***Author contributions.*** C. C., O. G., T. C. H., and H. L. designed the study. T. F. S., N. A., B. M., and I. S. collected the data. M. D. performed the analysis. T. F. S., C. C., O. G., K. G., T. C. H., M. L. F., L. O., P. V. d. S., and H. L. interpreted the data. All authors reviewed and commented on a draft version of the manuscript and gave their final approval for it to be submitted for publication.

***Acknowledgements.*** We thank the study participants. We also acknowledge the investigators and their clinical teams for their contribution to the study and their support and care of participants. We thank the global and regional clinical teams of GSK Vaccines for their contribution to the study, the research and development group for analyses of laboratory work, the scientific writers for clinical protocol and clinical report writing, the safety team for their input on safety analysis, and the statistician team for input on statistical analysis. We also thank Myriam Wilbaux (XPE Pharma & Science, Belgium; GSK Vaccines) for publication management and Kurt Liittschwager (4Clinics, France) for drafting the manuscript.

***Financial support.*** This work was supported by GSK Biologicals, which was involved in all stages of the study conduct and analysis and also funded all costs associated with the development and the publishing of the present manuscript.

***Potential conflicts of interest.*** C. C., M. D., K. G., M. L. F., L. O., and P. V. d. S. are employees, and O. G., T. C. H., and H. L. are former employees of the GSK group of companies. C.C., O.G., T. C. H., L. O., and H. L. hold shares in the GSK group of companies as part of their actual or former employee remuneration. T. C. H. is the coinventor of a patent application related to the vaccine used in this study. T.C.H. is currently paid as a consultant for GSK and that H.L. is employed by Pfizer Inc and holds stock as part of his remuneration. T. F. S. reports receiving personal fees from GSK. All other authors report no potential conflicts. All authors have submitted the ICMJE Form for Disclosure of Potential Conflicts of Interest. Conflicts that the editors consider relevant to the content of the manuscript have been disclosed.

Presented in part: IDWeek 2016 Congress, New Orleans, Louisiana, 26–30 October 2016.
