# Supplementary figures and images for: Immunogenicity and Safety of an Adjuvanted Herpes Zoster Subunit Vaccine Coadministered With Seasonal Influenza Vaccine in Adults Aged 50 Years or Older
Source: J Infect Dis. 2017 Sep 26;216(11):1352–61. doi: 10.1093/infdis/jix481 (PMC5853904; doi:10.1093/infdis/jix481)

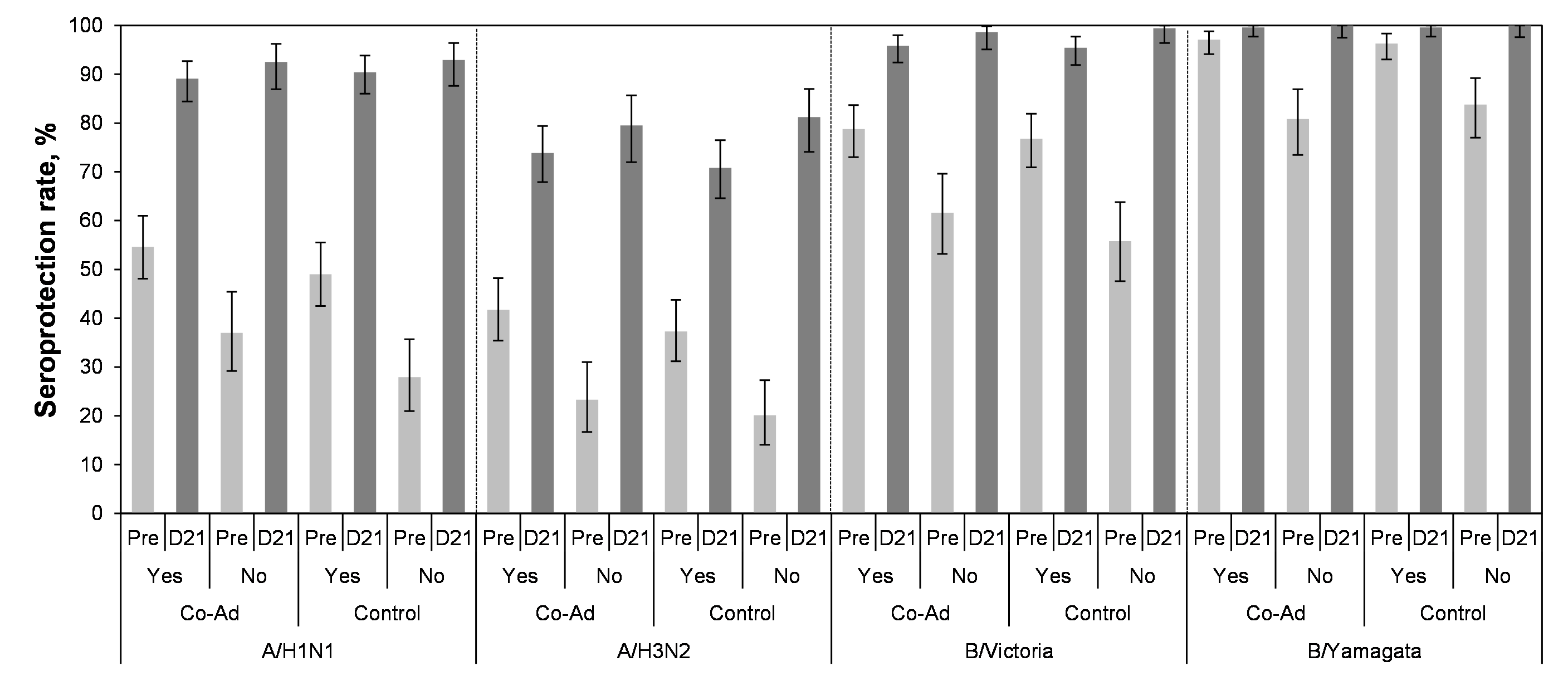

Supplement: Supplementary Figure S1 [file jix481_suppl_supplementary_figure_s1.png]

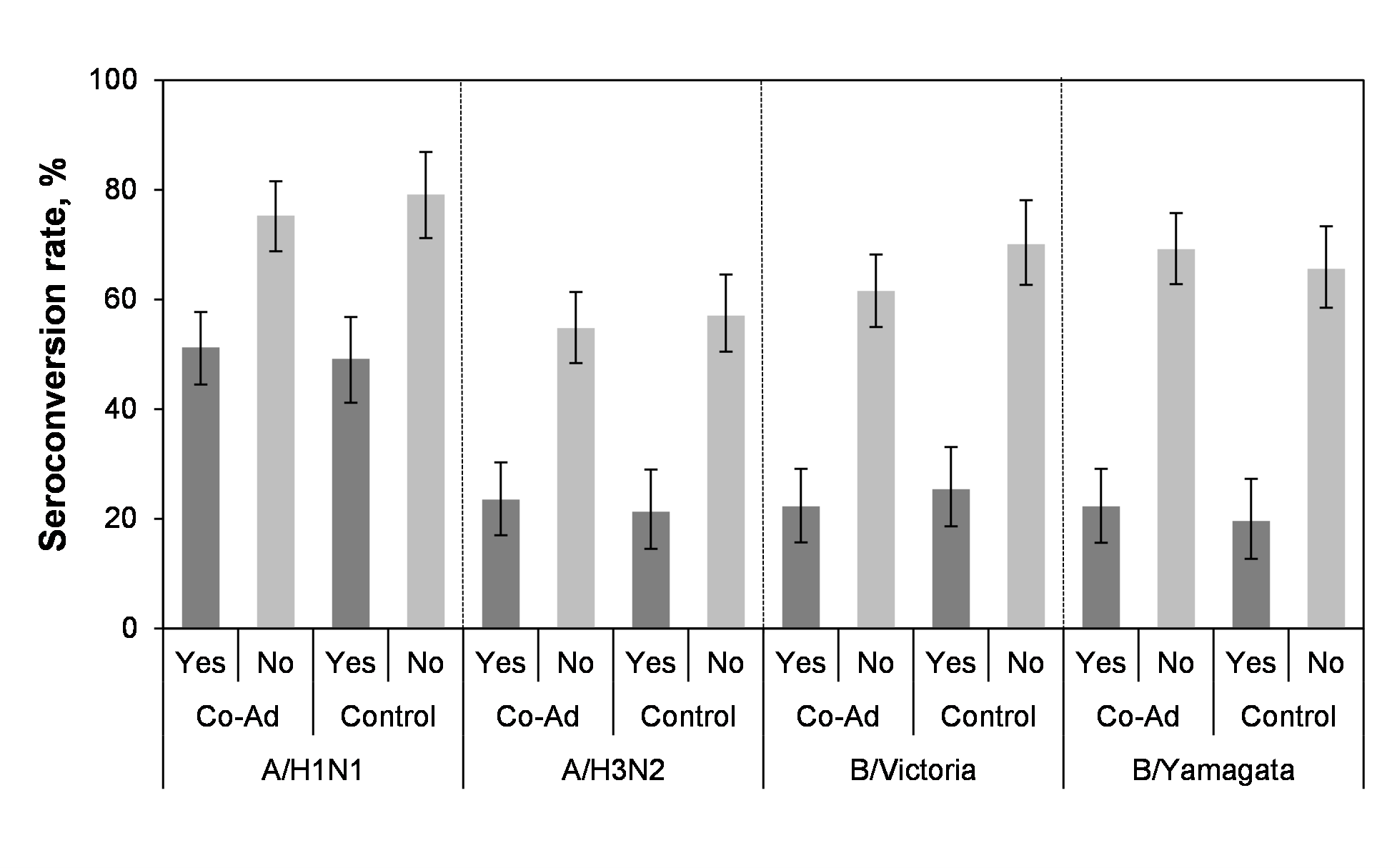

Supplement: Supplementary Figure S2 [file jix481_suppl_supplementary_figure_s2.png]
