# Supplementary material for: Immunogenicity and Safety of an Adjuvanted Herpes Zoster Subunit Vaccine Coadministered With Seasonal Influenza Vaccine in Adults Aged 50 Years or Older
Source: J Infect Dis. 2017 Sep 26;216(11):1352–61. doi: 10.1093/infdis/jix481 (PMC5853904; doi:10.1093/infdis/jix481)
Supplement: Patient Highlight Section [file jix481_suppl_patienthighlightsection.docx]

**Patient Highlight Section**

What is the context?

Varicella-zoster virus, the virus that causes chickenpox, remains inactive in the body after the initial infection. Reactivation of this virus later in life can result in herpes zoster or shingles typically causing a painful rash. The risk of shingles increases with age and is highest in adults ≥50 years of age. Shingles can also result in a condition called postherpetic neuralgia in which pain can last for months after the rash has resolved. A non-live candidate vaccine (HZ/su) against shingles has been recently developed and is currently undergoing regulatory review.

What is new?

This clinical trial was conducted to assess the immunologic and safety impact of giving the first dose of HZ/su at the same time as a seasonal vaccine against four strains of the influenza virus (IIV4) in adults aged 50 years or older. During this study the immune responses were similar between people who received the first dose of HZ/su at the same time as IIV4 and those who received HZ/su alone. Local reactions, like injection-site pain, redness and swelling, were observed similar whether subjects received both vaccines at the same time or not. Incidence of general reactions tended to be somewhat higher when the vaccines were administered on the same day compared to administration of a single vaccine. The most common general reactions were headache, myalgia, and fatigue. There were no serious adverse events or autoimmune diseases attributed to the studied vaccines.

What is the impact?

This study is important as it supports the concomitant administration of HZ/su and seasonal influenza vaccine at a single clinical visit.
